# Detection of HPV DNA in paraffin-embedded cervical samples: a comparison of four genotyping methods

**DOI:** 10.1186/s12879-015-1281-5

**Published:** 2015-11-25

**Authors:** Felipe A. Castro, Jill Koshiol, Wim Quint, Cosette M. Wheeler, Maura L. Gillison, Laurence M. Vaughan, Bernhard Kleter, Leen-Jan van Doorn, Anil K. Chaturvedi, Allan Hildesheim, Mark Schiffman, Sophia S. Wang, Rosemary E. Zuna, Joan L. Walker, S. Terence Dunn, Nicolas Wentzensen

**Affiliations:** Division of Cancer Epidemiology and Genetics, National Cancer Institute, Bethesda, MD USA; DDL, Diagnostic Laboratory, Rijswijk, The Netherlands; Viral Oncology Program, The Ohio State University Comprehensive Cancer Center, Columbus, OH USA; Department of Pathology, School of Medicine, University of New Mexico, Albuquerque, NM USA; BD Diagnostics, Sparks, MD USA; Division of Cancer Etiology, Department of Population Sciences, Beckman Research Institute and the City of Hope, Duarte, CA USA; Departments of Pathology and Obstetrics and Gynecology, University of Oklahoma Health Sciences Center, Oklahoma City, OK USA; Division of Cancer Epidemiology & Genetics, NCI Shady Grove, 9609 Medical Center Drive 7E114, Rockville, MD 20850 USA

**Keywords:** Genotyping, Formalin-fixed paraffin-embedded tissues, Cervical intraepithelial lesions, Exfoliated-cell

## Abstract

**Background:**

Identification of human papillomavirus (HPV) DNA in cervical tissue is important for understanding cervical carcinogenesis and for evaluating cervical cancer prevention approaches. However, HPV genotyping using formalin-fixed, paraffin-embedded (FFPE) tissues is technically challenging. We evaluated the performance of four commonly used genotyping methods on FFPE cervical specimens conducted in different laboratories and compared to genotyping results from cytological samples.

**Methods:**

We included 60 pairs of exfoliated-cell and FFPE specimens from women with histologically confirmed cervical intraepithelial lesions grade 2 or 3. Cytology specimens were genotyped using the Linear Array assay. Four expert laboratories processed tissue specimens using different preparation methods and then genotyped the resultant sample preparations using four different HPV genotyping methods: SPF_10_-PCR DEIA LiPA_25_ (version 1), Inno-LiPA, Linear Array and the Onclarity assay. Percentage agreement, kappa statistics and McNemar’s chi-square were calculated for each comparison of different methods and specimen types.

**Results:**

Overall agreement with respect to carcinogenic HPV status for FFPE samples between different methods was: 81.7, 86.7 and 91.7 % for Onclarity versus Inno-LiPA, Linear Array and SPF-LiPA_25_, respectively; 81.7 and 85.0 % for Linear Array versus Inno-LiPA and SPF-LiPA_25_, respectively; and 86.7 % for SPF-LiPA_25_ versus Inno-LiPA. Type-specific agreement was >88.3 % for all pair-wise comparisons. Comparisons with cytology specimens resulted in overall agreements from 80 to 95 % depending on the method and type-specific agreement was >90 % for most comparisons.

**Conclusions:**

Our data demonstrate that the four genotyping methods run by expert laboratories reliably detect HPV DNA in FFPE specimens with some variation in genotype-specific detection.

**Electronic supplementary material:**

The online version of this article (doi:10.1186/s12879-015-1281-5) contains supplementary material, which is available to authorized users.

## Background

The causal role of HPV in cervical carcinoma has been well established. Persistent infection with one of 13–15 oncogenic types of human papillomavirus (HPV) is a necessary cause of cervical cancer [1, 2]. Diagnosis of HPV infection is usually performed from cervical cytology specimens and used in natural history studies, implementation of screening programs, and follow-up of vaccination studies [3, 4]. However, cytology specimens often contain larger numbers of HPV infections [5], most of which are thought to be transient infections, whereas it is assumed that a lesion is caused by one genotype [6, 7]. Thus, identification of HPV in tissue specimens is important to ascertain the causal type involved in HPV-related carcinogenesis.

Detection and classification of HPV infection through polymerase chain reaction (PCR)-based methods has been successfully implemented in cervical cytology specimens [8]. Genotyping methods for HPV vary by target sequence and amplicon size. Most assays target the L1 gene and some the viral oncogenes E6/E7. Amplicon sizes range from 65 base pairs (SPF10 primers) to 450 base pairs (PGMY09/11 primers). Standard genotyping methods for cytology specimens often cannot be easily applied to tissue specimens. Particularly, formalin fixation may cause extensive DNA damage, including cross-linking and fragmentation [9, 10]. Consequently, it has been reported that successful amplification of HPV sequences from archival FFPE specimens is inversely correlated to the length of the amplicon of the PCR method and that specimen age may contribute to degradation [11, 12]. Furthermore, differences in sample processing and DNA extraction of FFPE materials may explain discrepancies observed in the performance of specific genotyping methods for FFPE specimens [13–15]. Several recent reports suggest that the use of robust extraction methods can improve the performance of nucleic acid tests when applied to fixed specimen types [16–18].

Given the importance of accurately identifying HPV present in FFPE materials and the lack of studies comparing HPV assays in tissue specimens, we evaluated HPV genotyping methods in FFPE specimens using four HPV genotyping methods, representing frequently-used assays with well-documented performance in cervical specimens. The laboratories participating in this comparison have long-standing experience in HPV genotyping and they have participated in many inter-laboratory comparisons, including WHO HPV LabNet proficiency testing.

## Methods

### Study population

The materials for this study were obtained from the Study to Understand Cervical Cancer Early Endpoints and Determinants (SUCCEED), a cross-sectional study including women referred to the University of Oklahoma from November 2003 to September 2009 for abnormal cervical screening results. SUCCEED design and methodology, including the details on enrollment, questionnaire data, HPV DNA genotyping, histology, and cytology procedures, have been described in depth elsewhere [19, 20]. Participants (median age 30.0 years) signed informed consent, completed interviewer-administered, standardized questionnaires and provided liquid-based cytology specimens for ThinPrep Pap and HPV genotyping. According to management guidelines, most histologically confirmed high-grade lesions diagnosed as cervical intraepithelial neoplasia grade 2 (CIN2) and all CIN3 were treated by loop electrosurgical excision procedure (LEEP) of the transformation zone.

For the present study, we selected 60 women diagnosed with CIN2+ (29 CIN2 and 31 CIN3) for whom tissue blocks were available and who had cytology-based HPV DNA genotyping results. The median duration between cytology sampling and LEEP was 27.5 days. In 26 women, cytology and LEEP were done on the same day.

Institutional Review Board approval for this study was provided by University of Oklahoma and the U.S. National Cancer Institute.

### HPV genotyping of cytology specimens

HPV detection and genotyping in cytology specimens was done using the Linear Array HPV Genotyping Test (Roche Molecular Diagnostics), as previously described [19, 20]. Briefly, the Linear Array assay is a type-specific, PGMY09/11 *L1* primer PCR-based assay for 37 HPV types, 13 high- and 24 low-risk HPV types (6, 11, 16, 18, 26, 31, 33, 35, 39, 40, 42, 45, 51, 52, 53, 54, 55, 56, 58, 59, 61, 62, 64, 66, 67, 68, 69, 70, 71, 72, 73, 81, 82, 83, 84, and 89). HPV 52 is not determined directly by a type-specific probe but rather by a probe that cross hybridizes with HPV 33, 35, 52, and 58. The presence of HPV 52 was inferred only if the cross-reactive probe was hybridized but there was no hybridization detected for the HPV 33, 35 and 58 type-specific probes. Notably, concurrent infections of type 52 with the three other types cannot be detected. The procedure followed recommendations of the manufacturer with the variations that DNA was isolated from 1 mL of exfoliated cells in PreservCyt using Qiagen DNeasy Blood and Tissue Kit, 10 μL of template DNA was amplified, and the amplified products were hybridized and detected using an automated Auto-line probe assay (LiPA) staining system using 2.5 mL of each reagent per strip. The Linear Array results on the strips were evaluated by two independent observers. An unambiguous, continuous band was judged to indicate a positive result.

### Tissue sectioning

A series of 4-μm-thick tissue sections was cut from each paraffin block. The first and last sections were stained with hematoxylin and eosin (H&E) to confirm the diagnosis of CIN2+. Each participating laboratory received two unstained 4um sections for HPV genotyping. Standard measures to avoid cross-contamination were taken during tissue sectioning and processing.

### HPV genotyping of tissue specimens

One of four HPV genotyping methods was used to genotype tissue specimens in each specific laboratory: 1) the The BD Onclarity™ HPV Assay (denoted as “Onclarity”); 2) the Inno-LiPA system (denoted as “Inno-LiPA”); 3) the PGMY09/11 Linear Array (denoted as “Linear Array”); and 4) SPF_10_-DEIA and LiPA_25_ (denoted as “SPF-LiPA_25_”). The main features of each HPV method are listed in Table 1 and described below. All FFPE sections, including those that tested HPV-negative, were positive for human DNA controls

**Table 1:** Formalin-fixed and paraffin embedded (FFPE) specimen processing, cytology specimens and human papillomavirus (HPV) testing methods

	Cytology specimens	HPV genotyping methods/protocol for FFPE specimens			
Characteristics	Linear array	Onclarity	Inno-LiPA	Linear array	SPF-LiPA_25_
Deparaffinization	NA	No	Yes	No	No
DNA extraction	Crude extraction	One-step heat / chemical lysis + FOX™ magnetic particle DNA purification	Xylene, proteinase K, phenol-chloroform extraction, ethanol precipitation	Crude extraction	Crude extraction
HPV testing	PGMY09/11 Linear Array	The BD Onclarity™HPV Assay	Inno-LiPA	PGMY09/11 Linear Array	SPF_10_-DEIA, LiPA_25_(version 1)
Probes for oncogenic types detectable	16, 18, 31, 33, 35, 39, 45, 51, 56, 58, 59, 66, 68 and 52/33/35/58	16, 18, 31, 45, 51, 52, 33/58, 39/68/35, and 59/ 56/66	16, 18, 31, 33, 35, 39, 45, 51, 52, 56, 58, 59, 66, and 68	16, 18, 31, 33, 35, 39, 45, 51, 56, 58, 59, 66, 68, and 52/33/35/58	16, 18, 31, 33, 35, 39, 45, 51, 52, 56, 58, 59, 66 and 68/73
Amplicon length (bp)	450	79-137	65	450	65

Onclarity, The BD Onclarity™ HPV Assay; Inno-LiPA, The Inno-LiPA system; Linear Array, The PGMY09/11 Linear Array, and SPF-LiPA_25_, The SPF_10_-DEIA, LiPA_25_(version 1) NA, not applicable; HPV 52 is positively identified in the linear Array when the presence of HPV 33, 35, and 58 is excluded

### Onclarity assay

The BD Onclarity™ HPV Assay (BD Diagnostics, Sparks, USA) is a real-time PCR assay that detects type-specific *E6* and *E7* genomic DNA. It simultaneously detects all 14 high-risk HPV types, and can provide genotyping information on six individual genotypes (HPV 16, 18, 31, 45, 51 and 52), reporting the remaining HPV types in three distinct groups (33 and 58; 56, 59 and 66; and 35, 39 and 68). Each FFPE patient sample was extracted using the automated workflow on the Viper™ LT system. The section was combined with 0.5 mL of distilled water and added directly to a tube with pierceable cap containing a proprietary diluent. The sample was then lysed directly using the Viper™ LT Pre-warm station before being transferred onto the deck of the instrument where it underwent automated sample processing and PCR detection. Briefly, the DNA was extracted using BD FOX™ magnetic particles and the eluate-containing DNA was used to set up three PCR genotyping reactions: G1 detects HPV 16, HPV 18 and HPV 45 plus the internal beta globin control; G2 detects HPV 31, HPV 33_58 and HPV 56_59_66 plus the internal beta globin control; G3 detects HPV 51, HPV 52 and HPV 35_39_68 plus the internal beta globin control. After 40 PCR cycles, any Ct score for a specific genotype and/or the internal beta globin control was considered positive for that channel.

### Inno-LiPA assay

DNA was purified from paraffin-embedded material as previously described [21]. Purified DNA was evaluated for the presence of HPV DNA by use of the Inno-LiPA assay (Innogenetics, Gent, Belgium), an assay that utilizes the SPF_10_ consensus primer system to amplify a 65 bp fragment of the L1 region of HPV, followed by reverse line blot hybridization to HPV type-specific immobilized probes for 18 high-risk/ possibly high-risk (16, 18, 25, 31, 33, 35, 39, 45, 51, 52, 53, 56, 58, 59, 66, 68, 73, 82) and 7 low-risk (6, 11, 40, 43, 44, 54, 70) HPV types. In addition to the internal assay control, testing for human DNA was conducted from all specimens (ERV3). The line probe assays are evaluated by two independent observers and were adjudicated by a third observer when different results are reported, which did not occur in this study.

### Linear array assay

Without removal of paraffin wax, the tissue sections obtained for HPV genotyping were resuspended (50–125 μl) in 10 mM Tris pH 8.0 containing 1 mm EDTA, 0.1 % Laureth-12 and 1 mg/ml proteinase K (PK) and digested with shaking at 65 °C for 4 h followed by overnight at 37 °C. Prior to polymerase chain reaction (PCR)-based HPV genotyping, PK was inactivated at 95 °C for 15 min. Microfuge tubes were immediately centrifuged briefly at 13,000 X g while the paraffin-wax was liquefied and an aqueous-wax interface formed upon cooling. Two and five microliters of the aqueous digest from each tissue specimen were used for genotyping with the LINEAR ARRAY HPV Genotyping Test (HPV LA; Roche Diagnostics, Indianapolis, Indiana USA). The LA HPV Genotyping Test is a qualitative test for 37 HPV genotypes incorporating selective PCR amplification with biotinylated PGMY 09/11 L1 region consensus primers and colorimetric detection of amplified products bound to immobilized HPV genotype –related oligonucleotide probes on a LINEAR ARRAY HPV genotyping strip. PGMY-based HPV genotyping with the HPV LA have been previously reported in detail [22, 23]. Using the Roche HPV LA detection kit, hybridizations were automated using Tecan ProfiBlot-48 robots (Tecan, Austria) as previously described [24]. Two independent readers interpreted the presence of HPV genotypes using a reference template provided by the manufacturer. Any discrepancies identified between the two readers were adjudicated by a third review.

### SPF-LiPA_25_ assay

Total DNA was isolated from FFPE tissue material by proteinase K treatment, 250 μl proteinase K lysis buffer was added and incubated at 56 °C for 16–24 h. Proteinase K was heat-inactivated by incubation at 96 °C for 10 min. Each DNA isolation run and PCR run contained HPV positive and negative controls.

Specimens were tested for HPV DNA by PCR amplification/typing using the HPV SPF_10_ PCR DEIA and LiPA_25_ version 1 assay (Labo Biomedical Products, Rijswijk, The Netherlands) based on licensed Innogenetics technology [25, 26]. The SPF_10_ PCR primer set detects a broad spectrum of HPV genotypes by amplification of a small fragment of 65 bp from the *L1* region of HPV. Reverse primers contain a biotin label at the 5’ end, enabling capture of the reverse strand onto streptavidin-coated microtiter plates. Captured amplimers are then denatured by alkaline treatment, and detected by a defined cocktail of digoxigenin-labeled probes, allowing detection of a broad spectrum of HPV genotypes. This method is designated an HPV DNA enzyme immunoassay (DEIA), and the results are an optical density value. When a sample is HPV positive by DEIA, the same SPF_10_ amplimer is also used to identify the HPV genotype by reverse hybridization to the LiPA_25_ genotyping strip (version 1). This line probe assay contains probes for 25 different HPV genotypes (i.e., HPV types 6, 11, 16, 18, 31, 33, 34, 35, 39, 40, 42, 43, 44, 45, 51, 52, 53, 54, 56, 58, 59, 66, 68/73, 70, and 74). The test results, purple lines on a LiPA strip are visually scored by two independent readers. In case of a discrepant line score a third reader determines the final outcome. In this study there were no different scores.

In SPF10 DEIA positive samples the presence of HPV 16 and 18 was also tested by type-specific (TS) PCR primer sets. TS16 and TS18 amplimers were detected by DEIA, similar to the method for SPF10 amplimer detection. The final genotyping result is the outcome of the testing algorithm [27]. As a result of this algorithm HPV16 and HPV 18 were only detected by type specific PCR three times and one time, respectively. These types were found in the presence of another HPV type. The SPF10 LiPA assay does not use an internal human DNA control. Two specimens were negative for all HPV types and 10fold dilution with and without spiking using HPV16 as a target did not indicate PCR inhibition.

### Statistical analyses

Analyses were limited to 14 HPV types classified as carcinogenic (16, 18, 31, 33, 35, 39, 45, 51, 52, 56, 58, 59, 66, 68) [2] and samples positive for other HPV types were considered as negative for HPV. We calculated overall and type-specific prevalence for high-risk HPV types detected in exfoliated cells by Linear Array and in FFPE specimens across all different methods.

To evaluate the performance of each method in FFPE tissue samples, we compared FFPE HPV genotyping results between different HPV methods. However, pair-wise comparisons were restricted to those carcinogenic types for which a specific probe was available across all of the different methods. Thus, comparison across all four assays was possible for types 16, 18, 31, 45, 51 and 52, and across 3 assays (Inno-LiPA, Linear Array and SPF-LiPA_25_) for types 33, 35, 39, 56 and 58, and 66. HPV type 68 genotyping was only compared between Linear Array and Inno-LiPA. HPV genotyping concordance was tested by calculating overall and type-specific percentages of agreement and kappa values. McNemar’s chi-square was calculated to test for significance (p < 0.05) in the pattern of disagreement.

An indirect analysis that may allow a further evaluation of the performance of these four genotyping methods in FFPE samples is compare FFPE genotyping results with those of their paired cytological sample. HPV genotyping results from FFPE specimens for each of the four assays was compared to results from cytology specimens as described above, and by creating three categories of agreement: (i) Identical: when the same number and type of HPV was identified in both specimens; (ii) Compatible: when at least one HPV type was found in common; and (iii) Discrepant: when no type was identified in common or the FFPE sample was HPV negative. This classification was performed, first by including all single and combined HPV type probes available in each of the assays and second, by including only single probes.

## Results

### Overall and type-specific HPV prevalence

All 60 exfoliated cell specimens were positive for at least one of the 14 carcinogenic HPV genotypes tested by Linear Array (Table 2). The most prevalent types were HPV16 (53.3 %), 18 (15.0 %), 31 and 45 (both 13.3 %). Multiple HPV types were found in 35 % of the exfoliated cell specimens.

**Table 2:** Human papillomavirus (HPV) genotyping results for paired cytology and tissue specimens^a^ by genotyping method (%)

	Cytology	FFPE samples			
HPV, type	Linear Array	Onclarity	Inno-LiPA	Linear Array	SPF-LiPA_25_
Any oncogenic^b^	60 (100.0)	54 (90.0)	51 (85.0)	48 (80.0)	57 (95.0)
Single probes					
HPV 16	32 (53.3)	26 (43.3)	25 (41.7)	24 (40.0)	29 (48.3)
HPV 18	9 (15.0)	8 (13.3)	7 (11.7)	5 (8.3)	8 (13.3)
HPV 31	8 (13.3)	7 (11.7)	7 (11.7)	6 (10.0)	7 (11.7)
HPV 33	2 (3.3)	NA	2 (3.3)	2 (3.3)	2 (3.3)
HPV 35	1 (1.7)	NA	0	0	0
HPV 39	4 (6.7)	NA	2 (3.3)	2 (3.3)	2 (3.3)
HPV 45	8 (13.3)	5 (8.3)	2 (3.3)	3 (5.0)	5 (8.3)
HPV 51	6 (10.0)	1 (1.7)	2 (3.3)	1 (1.7)	2 (3.3)
HPV 52	NA	5 (8.3)	3 (5.0)	NA	6 (10.0)
HPV 56	4 (6.7)	NA	1 (1.7)	1 (1.7)	1 (1.7)
HPV 58	5 (8.3)	NA	3 (5.0)	3 (5.0)	3 (5.0)
HPV 59	2 (3.3)	NA	0	2 (3.3)	1 (1.7)
HPV 66	4 (6.7)	NA	0	0	1 (1.7)
HPV 68	2 (3.3)	NA	0	1 (1.7)	NA
Combined probes					
52/33/35/58^c^	6 (10.0)	NA	NA	2 (3.3)	NA
33/58	NA	5 (8.3)	NA	NA	NA
35/39/68	NA	3 (5.0)	NA	NA	NA
56/59/66	NA	4 (6.7)	NA	NA	NA
68/73	NA	NA	NA	NA	2 (3.3)
Multiple infections^d^	21(35.0)	10 (16.6)	3 (5.0)	4 (6.7)	11 (18.3)

^a^ Genotyping results for 60 paired cytology- and formalin-fixed and paraffin embedded (FFPE) specimens in the SUCCEED study; Onclarity, The BD Onclarity ™ HPV Assay; Inno-LiPA, The Inno-LiPA system; Linear Array, The PGMY09/11 Linear Array, and SPF-LiPA_25_, The SPF_10_-DEIA, LiPA_25_ (version 1); ^b^ includes a specimen positive for any of the 14 HPV types; ^c^ HPV 52 was positively identified by the Linear array test when the presence of HPV 33, 35, and 58 was excluded; ^d^ Multiple infections included individual HPV types or combined types; NA, not applicable

Eighty percent or more of the FFPE specimens were positive for at least one HPV type in each of the four methods (Table 2). Type-specific positivity ranged across different HPV methods from 0 % (for HPV 35, 59, 66, and 68 in some of the tested methods) to 48.3 % (for HPV 16 using the SPF-LiPA_25_ method). The SPF-LiPA_25_ assay identified more types at both the overall and the type-specific level. Detection of multiple types was rare among FFPE specimens by all methods (from 5.0 % in the Inno-LiPA to 18.3 % in the SPF-LiPA_25_). There was a notable difference in the detection rate of multiple types between tests with the Onclarity and SPF-LiPA_25_ method detecting 2–3 times more multiple types than those detected by the Inno-LiPA and Linear Array methods (Table 2). Genotyping results for each of the participants are provided in Additional file 1: Table S1. Only one specimen was negative across all methods. In three cases, Onclarity detected types that were not found with any of the other assays. Six specimens were repeated in three labs; only one repeat results showed a partial discrepancy (Additional file 2: Table S2).

Multiple HPV genotypes were detected in 21 of the 60 exfoliated cell specimens. In the corresponding FFPE specimen multiple genotypes were only detected in eight specimens by at least one of the four different genotyping assays. This observation can be explained by the fact that exfoliated cell specimens cover the complete cervix area, whereas FFPE specimens are taken at a specific site of the cervix epithelium.

### Agreement between tissue-based HPV testing across HPV methods

Overall concordance between tissue-based HPV results across laboratory methods was good (Table 3); the overall percent agreement ranged from 81.7 for Inno-LiPA compared with Linear Array and with Onclarity to 91.7 % for SPF-LiPA_25_ compared with Onclarity. The number of samples with HPV types detected was lower when samples were genotyped by Linear Array compared with the number of positive samples obtained by SPF-LiPA_25_ (McNemar’s p-value =0.004). Percentage of agreement for the two most common HPV types, 16 and 18, ranged from 88.3 % (SPF-LiPA_25_ vs. Onclarity) to 95 % (Onclarity vs. Linear Array) for HPV 16 and from 95 % (SPF-LiPA_25_ vs. Linear Array and Onclarity vs. Linear Array) to 98.3 % (Onclarity vs. Inno-LiPA and SPF-LiPA_25_ vs. Inno-LiPA) for HPV 18, but none of the differences were statistically significant. Summarized across all types, the percent identical, compatible and discrepant results for each assay combination is shown in Additional file 3: Table S3.

**Table 3:** Human papillomavirus (HPV) genotyping agreement for tissue specimens* tested with four different genotyping methods

HPV	Method 1		Method 2		Percent^a^	Kappa			
Type	Name	Positive	Name	Positive	agreement	Value	95 % CI		*P*
Oncogenic^b^									
Any	Onclarity	54	Inno-LiPA	51	81.7	0.2	−0.15	0.48	0.5
Any	Onclarity	54	Linear Array	48	86.7	0.5	0.19	0.78	0.07
Any	Onclarity	54	SPF-LiPA_25_	57	91.7	0.4	−0.01	0.82	0.4
Any	Linear Array	48	Inno-LiPA	51	81.7	0.4	0.07	0.67	0.5
Any	Linear Array	48	SPF-LiPA_25_	57	85.0	0.3	0.05	0.65	0.004
Any	SPF-LiPA_25_	57	Inno-LiPA	51	86.7	0.3	−0.06	0.62	0.07
By single probe									
16	Onclarity	26	Linear Array	24	90.0	0.79	0.64	0.95	0.7
16	Onclarity	26	Inno-LiPA	25	91.7	0.83	0.69	0.97	1.0
16	Linear Array	24	Inno-LiPA	25	95.0	0.9	0.78	1.0	1.0
16	SPF-LiPA_25_	29	Onclarity	26	88.3	0.77	0.6	0.93	0.5
16	SPF-LiPA_25_	29	Inno-LiPA	25	90.0	0.8	0.65	0.95	0.2
16	SPF-LiPA_25_	29	Linear Array	24	91.7	0.83	0.69	0.97	0.1
18	Onclarity	8	Inno-LiPA	7	98.3	0.92	0.78	1.0	1.0
18	Onclarity	8	Linear Array	5	95.0	0.74	0.47	1.0	0.3
18	Linear Array	5	Inno-LiPA	7	96.7	0.82	0.57	1.0	0.5
18	SPF-LiPA_25_	8	Linear Array	5	95.0	0.74	0.47	1.0	0.3
18	SPF-LiPA_25_	8	Inno-LiPA	7	98.3	0.92	0.78	1.0	1.0
18	SPF-LiPA_25_	8	Onclarity	8	96.7	0.86	0.66	1.0	1.0
31	Onclarity	7	Inno-LiPA	7	96.7	0.84	0.62	1.0	1.0
31	Onclarity	7	Linear Array	6	98.3	0.91	0.75	1.0	1.0
31	Linear Array	6	Inno-LiPA	7	98.3	0.91	0.75	1.0	1.0
31	SPF-LiPA_25_	7	Linear Array	6	98.3	0.91	0.75	1.0	1.0
31	SPF-LiPA_25_	7	Onclarity	7	96.7	0.84	0.62	1.0	1.0
31	SPF-LiPA_25_	7	Inno-LiPA	7	100	NA	NA	NA	NA
33	Linear Array	2	Inno-LiPA	2	100	NA	NA	NA	NA
33	SPF-LiPA_25_	2	Linear Array	2	100	NA	NA	NA	NA
33	SPF-LiPA_25_	2	Inno-LiPA	2	100	NA	NA	NA	NA
35	Linear Array	0	Inno-LiPA	0	NA	NA	NA	NA	NA
35	SPF-LiPA_25_	0	Inno-LiPA	0	NA	NA	NA	NA	NA
35	SPF-LiPA_25_	0	Linear Array	0	NA	NA	NA	NA	NA
39	Linear Array	2	Inno-LiPA	2	100	NA	NA	NA	NA
39	SPF-LiPA_25_	2	Inno-LiPA	2	100	NA	NA	NA	NA
39	SPF-LiPA_25_	2	Linear Array	2	100	NA	NA	NA	NA
45	Onclarity	5	Inno-LiPA	2	91.7	0.25	−0.19	0.69	0.4
45	Onclarity	5	Linear Array	3	93.3	0.47	0.03	0.91	0.6
45	Linear Array	3	Inno-LiPA	2	95.0	0.38	−0.18	0.93	1.0
45	SPF-LiPA_25_	5	Linear Array	3	96.7	0.73	0.38	1.0	0.5
45	SPF-LiPA_25_	5	Inno-LiPA	2	95.0	0.55	0.11	0.99	0.3
45	SPF-LiPA_25_	5	Onclarity	5	96.7	0.78	0.49	1.0	1.0
51	Onclarity	1	Inno-LiPA	2	98.3	0.66	0.04	1.0	1.0
51	Onclarity	1	Linear Array	1	100	NA	NA	NA	NA
51	Linear Array	1	Inno-LiPA	2	98.3	0.66	0.04	1.0	1.0
51	SPF-LiPA_25_	2	Linear Array	1	98.3	0.66	0.04	1.0	1.0
51	SPF-LiPA_25_	2	Onclarity	1	98.3	0.66	0.04	1.0	1.0
51	SPF-LiPA_25_	2	Inno-LiPA	2	100	NA	NA	NA	NA
52	Onclarity	5	Inno-LiPA	3	93.3	0.47	0.03	0.91	0.6
52	Onclarity	5	Linear Array	2	95.0	0.55	0.11	0.99	0.3
52	Linear Array	2	Inno-LiPA	3	98.3	0.79	0.4	1.0	1.0
52	SPF-LiPA_25_	6	Linear Array	2	93.3	0.47	0.05	0.9	0.1
52	SPF-LiPA_25_	6	Inno-LiPA	3	95.0	0.64	0.27	1.0	0.3
52	SPF-LiPA_25_	6	Onclarity	5	95.0	0.7	0.38	1.0	1.0
56	Linear Array	1	Inno-LiPA	1	100	NA	NA	NA	NA
56	SPF-LiPA_25_	1	Linear Array	1	100	NA	NA	NA	NA
56	SPF-LiPA_25_	1	Inno-LiPA	1	100	NA	NA	NA	NA
58	Linear Array	3	Inno-LiPA	3	100	NA	NA	NA	NA
58	SPF-LiPA_25_	3	Linear Array	3	100	NA	NA	NA	NA
58	SPF-LiPA_25_	3	Inno-LiPA	3	100	NA	NA	NA	NA
59	Linear Array	2	Inno-LiPA	0	NA	NA	NA	NA	NA
59	SPF-LiPA_25_	1	Linear Array	2	98.3	0.66	0.04	1.0	1.0
59	SPF-LiPA_25_	1	Inno-LiPA	0	NA	NA	NA	NA	NA
66	Linear Array	0	Inno-LiPA	0	NA	NA	NA	NA	NA
66	SPF-LiPA_25_	1	Inno-LiPA	0	NA	NA	NA	NA	NA
66	SPF-LiPA_25_	1	Linear Array	0	NA	NA	NA	NA	NA
68	Linear Array	1	Inno-LiPA	0	NA	NA	NA	NA	NA

* Genotyping agreement for 60 formalin-fixed and paraffin embedded (FFPE) specimens in the SUCCEED study; Onclarity, The BD Onclarity™ HPV Assay; Inno-LiPA, The Inno-LiPA system; Linear Array, The PGMY09/11 Linear Array, and SPF-LiPA_25_, The SPF_10_-DEIA, LiPA_25_ (version 1); p, p-value of the McNerman test; ^b^ Any, includes a specimen positive for any of the 14 HPV types; NA, not apply or cannot be calculated; HPV 52 was positive when there was a test negative for HPV 33, 35, and 58 individually but positive for the combined probe 52/33/35/58 (for Linear Array)

### Agreement between exfoliated-cell- and tissue-based HPV testing

Identical HPV results between cytology and FFPE were found in 53.3 % (Onclarity), 56.7 % (Inno-LiPA), 53.3 % (Linear Array), and 61.7 % (SPF-LiPA_25_) of the cases (Table 4); an additional 25.0 to 33.3 % were categorized as compatible HPV results and 5.0 to 21.7 % as discrepant results, meaning no type agreement or sample is HPV negative. Almost all discrepant results were related to a negative test result.

**Table 4:** Human papillomavirus (HPV) genotyping agreement for paired cytology and tissue specimens^a^ tested with different genotyping methods

Overall agreement	Onclarity			Inno-LiPA			Linear Array			SPF-LiPA_25_		
with cytology	N (%)			N (%)			N (%)			N (%)		
Identical	32 (53.3)			34 (56.7)			32 (53.3)			37 (61.7)		
Compatible	19 (31.7)			17 (28.3)			15 (25.0)			20 (33.3)		
Discrepant	9 (15.0)			9 (15.0)			13 (21.7)			3 (5.0)		
HPV type	%^a^	kappa	*p*	%^b^	kappa	*p*	%^a^	kappa	*p*	%^a^	kappa	*p*
Any oncogenic type^c^	90.0	NA		85.0	NA		80.0	NA		95.0	NA	
Single probe												
HPV 16	83.3	0.7	0.1	88.3	0.8	0.02	86.7	0.7	0.01	88.3	0.8	0.5
HPV 18	95	0.8	1.0	96.7	0.9	0.5	93.3	0.7	0.1	98.3	0.9	1.0
HPV 31	95	0.8	1.0	98.3	0.9	1.0	96.7	0.8	0.5	98.3	0.9	1.0
HPV 33	NA			100	1.0	NA	100	1.0	NA	100	1.0	NA
HPV 35	NA			98.3	NA		98.3	NA		98.3	NA	
HPV 39	NA			96.7	0.7	0.5	96.7	0.7	0.5	96.7	0.7	0.5
HPV 45	91.7	0.6	0.1	90.0	0.4	0.03	91.7	0.5	0.1	95.0	0.7	0.3
HPV 51	91.7	0.3	0.1	93.3	0.5	0.1	91.7	0.3	0.1	93.3	0.5	0.1
HPV 52^d^	98.3	0.9	1.0	95.0	0.6	0.3	93.3	0.5	0.1	96.7	0.8	1.0
HPV 56	NA			95.0	0.4	0.3	95.0	0.4	0.3	95.0	0.4	0.3
HPV 58	NA			96.7	0.7	0.5	96.7	0.7	0.5	96.7	0.7	0.5
HPV 59	NA			NA			100	1.0	NA	98.3	0.7	1.0
HPV 66	NA			NA			NA			95.0	0.4	0.3
HPV 68	NA			NA			98.3	0.7	1.0	NA		

^a^ Genotyping agreement for 60 paired cytology- and formalin-fixed and paraffin embedded (FFPE) specimens in the SUCCEED study; Onclarity, The BD Onclarity™ HPV Assay; Inno-LiPA, The Inno-LiPA system; Linear Array, The PGMY09/11 Linear Array, and SPF-LiPA_25_, The SPF_10_-DEIA, LiPA_25_ (version 1); Identical, same number and type identified; Compatible, at least one type in common identified; Discrepant, no type in common identified or FFPE sample HPV negative; ^a^ Only single probes, but including probe combined probe 52/33/35/58 for Linear Array;^b^ Percentage of overall agreement; ^c^ Includes a specimen positive for any of the 14 HPV types; ^d^ HPV 52 was positively identified in the linear Array test when the presence of HPV 33, 35, and 58 was excluded; *p*, p-value of the McNemar test; NA, not apply or cannot be calculated

The overall agreement of HPV status between exfoliated-cell and FFPE specimens across the four tested methods was 90 % for Onclarity, 85.0 % for Inno-LiPA, 80 % for Linear Array, and 95 % for SPF-LiPA_25_ (Table 4). When considering individual types, the percentage agreement ranged from 83.3 % (HPV16) to 98.3 (HPV52) for the Onclarity method, 88.3 % (HPV16) to 100 % (HPV33) for the SPF-LiPA_25_ and the Inno-LiPA methods, and from 86.7 % (HPV 16) to 100 % (HPV33 and 59) for the Linear Array method. Detection for HPV16 and 45 tested for the Inno-LiPA method and for HPV16 for the Linear Array were significantly lower in FFPE samples compared to detection in exfoliated specimens (McNemar’s p-value of 0.02 and 0.03, and 0.01, respectively).

## Discussion

Very few studies have evaluated the performance of two or more methods in parallel in FFPE specimens [28–30]. In the present study, we evaluated simultaneously four different methods that not only varied in the set of primers used, but also in the sample processing and DNA extraction protocols of the independent laboratories. Even with such marked differences in the protocols, we observed good overall performance for all methods in the detection of HPV in FFPE specimens.

With the exception of the Onclarity assay, the genotyping performance of these HPV methods has been previously evaluated in FFPE specimens. Our findings are very much in agreement with other reports indicating a good to excellent performance of SPF-LiPA_25_, Inno-LiPA and the Linear Array method. Previous studies evaluating the SPF-LiPA_25_ assay in FFPE and cytology specimens found an overall percentage agreements greater than 90 %[13, 15, 31]. The SPF-LiPA_25_ has been also compared with other methods, such as the Genomica assay (primers MY09/1) [29] and the Linear Array assay [28], with similar results. Although there were methodological differences in those studies (genotyping method used for cytology specimens, grade of cervical lesion and time window between the testing of the cytological and tissue specimens), they all reported good agreement between SPF_10_-LiPA_25_ and the other tests. The SPF-LiPA_25_ method uses the general primer set, designed as SPF_10_, which amplifies a fragment of only 65 bp of L1 region of the HPV genome [26]. This primer set has a high sensitivity for HPV detection, which makes it particularly suitable for assessing HPV in FFPE specimens.

Good performance for the Inno-LiPA method in FFPE versus cytology specimens has been also reported in previous studies. One Italian study compared the Inno-LiPA genotyping results from FFPE samples with the Linear Array results from the paired cytology specimens and reported an overall kappa value of 0.85 [14]. Similarly, a Slovenian study compared the performance of a real time PCR assay versus the Inno-LiPA method in 31 FFPE cervical cancer specimens, reporting 100 % genotype agreement [32]. The Inno-LiPA method also performed well in FFPE specimens from other HPV-related cancers, such as head and neck [33] and vulva [30, 34].

To our knowledge, no previous study has compared FFPE samples tested by Linear Array with cytological results by Linear Array or any other method. We saw similar HPV genotyping results for FFPE and cytology specimens tested by Linear Array (agreement 80 %). As mentioned above, Linear Array has been directly compared with SPF-LiPA_25_ and with Inno-LiPA in FFPE cervical [35] and vulvar specimens [30], respectively. Such studies showed good concordance between the methods for detecting any high-risk types. However, both previous studies [30, 35] and our study found that the sensitivity of Linear Array was lower compared to other methods. The lower sensitivity of Linear Array in FFPE samples could be attributed to DNA fragmentation; the Linear Array method targets an amplicon region much larger than other methods (450 bp), which can result in reduced amplification efficiency [36]. A limitation of Linear Array is the lack of an individual probe for HPV 52. Since HPV52 is detected in a mixed probe together with HPV-33, −35, or −58, multiple infections between HPV52 and one of these types cannot be detected.

Our study is the first one to test the Onclarity genotyping method using FFPE specimens. Previous studies have found that this method performs well in cytology specimens in comparison with other methods such as Hybrid Capture 2, Linear Array and Line Blot Assay [37]. Likewise a comprehensive study that compared seven genotyping methods in cytology specimens showed that Onclarity assay has a sensitivity of 95 % for detection of CIN3+ [38]. Thus, the good performance of Onclarity in FFPE specimens is consistent with previous studies using cytology specimens. The BD Onclarity™ HPV Assay (BD Diagnostics) uses real-time PCR to detect 14 HR HPV types, six of which are tested individually while the remaining HPV types are grouped (33_58), (56_59_66) and (35_39_68). The HPV typing using multiple types in a single channel prevented the evaluation of the type specific agreement for these combined types and may limit the use of the assay when individual genotyping of these combined types is required.

We observed that independent of the genotyping method used, overall HPV prevalence (any type versus none) in the FFPE specimens (between 80 to 95 % across methods) was lower than in cytology specimens (100 % with Linear Array), as has been found in some previous studies [7, 15, 31], but not others [13]. Although the sample size was too limited for a formal test, we did not see evidence of type-specific failures for any of the evaluated methods (Additional file 1: Table S1). We noted a difference in the detection of multiple types, with the Onclarity and SPF-LiPA_25_ tests detecting more multiple types than either the Inno-LiPA or Linear Array tests. This may be related to smaller target sequences of these two assays, or to the previously described reduced performance of select L1 primer designs in detecting multiple infections [39]. While a smaller amplicon size could potentially have a higher risk of cross-sample contamination, we did not observe any sign of contamination in our study.

DNA fragmentation and DNA-protein cross-linking by formaldehyde exposure, as well as the presence of paraffin, can impact both DNA yield and PCR amplification efficiency. Our study evaluated combinations of DNA extraction methods and HPV genotyping assays performed in different laboratories. Therefore, the differences observed in this study cannot be directly attributed to assay performance versus the DNA extraction approach used. However, generally, the differences between results from different laboratories were minor and seemed to be related mostly to amplicon size.

Strengths of the current analysis are the complete histological characterization of the selected specimens, and the short interval between cytological and histological specimen collection. A limitation of our study was the lack of parallel genotyping data from cytology specimens for all the tested genotyping methods.

## Conclusions

HPV genotyping from FFPE tissues showed high intra- and inter-laboratory reproducibility in our study; the use of any of the tested HPV genotyping methods in expert laboratories can provide reliable identification of the most important high risk HPV types in FFPE specimens. However, there remains concern that reliability of tissue-based genotyping may be worse in less experienced and less rigorous laboratories, as indicated by a WHO HPV laboratory comparison study including 29 centers across the world, which reported lower proficiency measures for HPV detection despite the use of standardized DNA samples [40], rather than whole tissue sections as in the current study. Thus, it can be expected that there is higher variation of proficiency for tissue-based genotyping in less experienced and less rigorous laboratories.

## Additional files

Additional file 1: Table S1. Human papillomavirus (HPV) genotyping results for 60 formalin-fixed and paraffin embedded (FFPE) specimens tested in four different laboratories compared to the paired cytology specimen tested by Linear Array. (DOC 101 kb) Additional file 2: Table S2. Human papillomavirus (HPV) genotyping reproducibility for 6 formalin-fixed and paraffin embedded (FFPE) specimens by genotyping method and the paired HPV cytological result in the SUCCEED study. (DOC 34 kb) Additional file 3: Table S3. Human papillomavirus (HPV) genotyping agreement for tissue specimens* tested with different genotyping methods. (DOC 31 kb)

## Abbreviations

HPV: Human papillomavirus
FFPE: Formalin-fixed, paraffin-embedded
PCR: Polymerase chain reaction
CIN: Cervical intraepithelial neoplasia
LEEP: Loop electrosurgical excision procedure
EDTA: Ethylenediaminetetraacetic acid
PK: Proteinase K
DEIA: DNA enzyme immunoassay
WHO: World Health Organization
