# Supplementary material for: Detection of HPV DNA in paraffin-embedded cervical samples: a comparison of four genotyping methods
Source: BMC Infect Dis. 2015 Nov 25;15:544. doi: 10.1186/s12879-015-1281-5 (PMC4660657; doi:10.1186/s12879-015-1281-5)
Supplement: Additional file 1: Table S1. — Human papillomavirus (HPV) genotyping results for 60 formalin-fixed and paraffin embedded (FFPE) specimens tested in four different laboratories compared to the paired cytology specimen tested by Linear Array. (DOC 101 kb) [file 12879_2015_1281_MOESM1_ESM.doc]

**Supplementary table 1. Human papillomavirus (HPV) genotyping results for 60 formalin-fixed and paraffin embedded (FFPE) specimens tested in four different laboratories compared to the paired cytology specimen tested by Linear Array**

|  | **Cytology specimens** | **FFPE specimens** | | | | | | | |
| --- | --- | --- | --- | --- | --- | --- | --- | --- | --- |
|  | **Linear Array** | **Onclarity** | | **Inno-LiPA** | | **Linear Array** | | **SPF-LiPA_25_** | |
| **ID** | **Type** | **Type** | Category | **Type** | Category | **Type** | Category | **Type** | Category |
| 1 | 16 | 16 | I | 16 | I | 16 | I | 16 | I |
| 2 | 16 | 16 | I | 16 | I | 16 | I | 16 | I |
| 3 | 16 | 16 | I | 16 | I | 16 | I | 16 | I |
| 4 | 16 | 16 | I | 16 | I | 16 | I | 16 | I |
| 5 | 16 | 16 | I | 16 | I | 16 | I | 16 | I |
| 6 | 16 | 16 | I | 16 | I | 16 | I | 16 | I |
| 7 | 16 | 16 | I | 16 | I | 16 | I | 16 | I |
| 8 | 16 | 16 | I | 16 | I | 16 | I | 16 | I |
| 9 | 16 | 16 | I | 16 | I | 16 | I | 16 | I |
| 10 | 16 | 16 | I | 16 | I | 16 | I | 16 | I |
| 11 | 16 | 16 | I | 16 | I | 16 | I | 16 | I |
| 12 | 16 | 16 | I | 16 | I | 16 | I | 16 | I |
| 13 | 16 | 16 | I | 16 | I | 16 | I | 16 | I |
| 14 | 16 | 16 | I | 16 | I | 16 | I | 16 | I |
| 15 | 16 | 16 | I | 16 | I | 16 | I | 16 | I |
| 16 | 16 | 45 | D | 16 | I | 16 | I | 16 | I |
| 17 | 16 | 16,35/39/68 | C | 16 | I | 16 | I | 16 | I |
| 18 | 16 | 18,31 | D | NEG | D | 16 | I | 16 | I |
| 19 | 16 | 16 | I | 16 | I | NEG | D | 16 | I |
| 20 | 16 | NEG | D | 16 | I | NEG | D | NEG | D |
| 21 | 16 | NEG | D | NEG | D | NEG | D | NEG | D |
| 22 | 18 | 18 | I | 18 | I | 18 | I | 18 | I |
| 23 | 18 | 18 | I | 18 | I | 18 | I | 18 | I |
| 24 | 18 | 18 | I | 18 | I | NEG | D | 18 | I |
| 25 | 18 | 16,18 | C | 18 | I | NEG | D | 18,52 | C |
| 26 | 31 | 31 | I | 31 | I | 31 | I | 31 | I |
| 27 | 31 | 31 | I | 31 | I | 31 | I | 31 | I |
| 28 | 31 | NEG | D | 31 | I | NEG | D | 16,31 | C |
| 29 | 45 | 16 | D | 45 | I | 45 | I | 45 | I |
| 30 | 45 | 45 | I | NEG | D | 45 | I | 45 | I |
| 31 | 45 | 45 | I | NEG | D | NEG | D | 45 | I |
| 32 | 45 | 45 | I | 45 | I | NEG | D | 16,45 | C |
| 33 | 45 | 45 | I | NEG | D | 45 | I | 45,68 ^a^ | C |
| 34 | 51 | 51 | I | 51 | I | 51 | I | 51 | I |
| 35 | 59 | 56/59/66 | C | NEG | D | 59 | I | 59 | I |
| 36 | 16,18 | 16,18 | I | 18 | C | 18 | C | 16,18 | I |
| 37 | 16,18 | 16,18 | I | 16, 18 | I | 16,18 | I | 16,18 | I |
| 38 | 16,18,31,39,56,66 | 35/39/68 | C | 39 | C | 39 | C | 39 | C |
| 39 | 16,31,35,45,52/33/35/58,59 | 31 | C | 31 | C | 31,59 | C | 16,31 | C |
| 40 | 16,39,51,52/33/35/58,58 | 33/58 | C | 39, 58 | C | 39,58 | C | 39,58 | C |
| 41 | 16,39,52/33/35/58 | 52 | C | 52 | C | 52 | C | 52 | C |
| 42 | 16,45 | 16 | C | 16 | C | 16 | C | 16 | C |
| 43 | 16,51,56 | 16 | C | 16 | C | 16 | C | 16 | C |
| 44 | 16,52/33/35/58,58 | 16 | C | 16 | C | 16 | C | 16 | C |
| 45 | 16,68 | 16 | C | 16 | C | 16 | C | 16 | C |
| 46 | 16,68 | 16,35/39/68 | C | 16 | C | 16,68 | I | 16,68^a^ | C |
| 47 | 18,51,52/33/35/58,56,58 | 33/58,56/59/66 | C | 51, 56 | C | 56 | C | 18,51,56 | C |
| 48 | 18,52/33/35/58 | 18,52 | I | 18 | C | 18 | C | 18 | C |
| 49 | 31,52/33/35/58 | 31,52 | I | 31 | C | 31 | C | 31,52 | I |
| 50 | 31,56 | 31,56/59/66 | C | 31 | C | 31 | C | 31 | C |
| 51 | 31,66 | 31 | C | 31 | C | 31 | C | 31 | C |
| 52 | 33,39,52/33/35/58 | 33/58 | C | 33 | C | NEG | D | 33 | C |
| 53 | 33,52/33/35/58 | 33/58 | C | 33 | I | 33 | I | 33 | I |
| 54 | 45,52/33/35/58,58 | NEG | D | 58 | C | 58 | C | 58 | C |
| 55 | 51,66 | NEG | D | NEG | D | NEG | D | 66 | C |
| 56 | 51,66 | 56/59/66 | C | NEG | D | NEG | D | NEG | D |
| 57 | 52/33/35/58 | 52 | I | 52 | I | 52 | I | 52 | I |
| 58 | 52/33/35/58 | NEG | D | 52 | I | NEG | D | 52 | I |
| 59 | 52/33/35/58 | 52 | I | NEG | D | NEG | D | 52 | I |
| 60 | 52/33/35/58,58 | 33/58 | C | 58 | I | 58 | I | 58 | I |

Onclarity, The BD Onclarity™ HPV Assay; Inno-LiPA , The Inno-LiPA system ; Linear Array , The PGMY09/11 Linear Array, and SPF-LiPA , The SPF_10_-DEIA, LiPA (version 1); ); I (Identical), same number and type identified ; C (Compatible), at least one type in common identified ; D (Discrepant), no type in common identified or FFPE sample HPV negative; HPV 52 was positively identified in the Linear Array when the presence of HPV 33, 35, and 58 was excluded; ^a^ Method SPF-LiPA genotyped HPV 68 and 73 together.
