# Supplementary material for: Detection of HPV DNA in paraffin-embedded cervical samples: a comparison of four genotyping methods
Source: BMC Infect Dis. 2015 Nov 25;15:544. doi: 10.1186/s12879-015-1281-5 (PMC4660657; doi:10.1186/s12879-015-1281-5)
Supplement: Additional file 2: Table S2. — Human papillomavirus (HPV) genotyping reproducibility for 6 formalin-fixed and paraffin embedded (FFPE) specimens by genotyping method and the paired HPV cytological result in the SUCCEED study. (DOC 34 kb) [file 12879_2015_1281_MOESM2_ESM.doc]

**Supplementary table 2. Human papillomavirus (HPV) genotyping reproducibility for 6 formalin-fixed and paraffin embedded (FFPE) specimens by genotyping method and the paired HPV cytological result in the SUCCEED study**

|  | **Cytology** | **FFPE samples** | | |
| --- | --- | --- | --- | --- |
| **Sample** | **Linear Array** | **SPF-LiPA_25_** | **Linear Array** | **Onclarity** |
| 1 | 16, 18, 31, 39, 56, 66 | 39 | 39 | 39/68/35 |
|  |  | 39 | 39 | 39/68/35 |
| 2 | 16, 52/33/35/58, 58 | 16 | 16 | 16 |
|  |  | 16 | 16 | 16 |
| 3 | 16 | 16 | 16 | 16 |
|  |  | 16 | 16 | 16,39/68/35 |
| 4 | 16 | 16 | 16 | 16 |
|  |  | 16 | 16 | 16 |
| 5 | 16 | 16 | 16 | 16 |
|  |  | 16 | 16 | 16 |
| 6 | 16, 45 | 16 | 16 | 16 |
|  |  | 16 | 16 | 16 |

Onclarity, The BD Onclarity™ HPV Assay; Linear Array , The PGMY09/11 Linear Array, and SPF-LiPA , The SPF_10_-DEIA, LiPA (version 1)
