# Supplementary material for: Detection of HPV DNA in paraffin-embedded cervical samples: a comparison of four genotyping methods
Source: BMC Infect Dis. 2015 Nov 25;15:544. doi: 10.1186/s12879-015-1281-5 (PMC4660657; doi:10.1186/s12879-015-1281-5)
Supplement: Additional file 3: Table S3. — Human papillomavirus (HPV) genotyping agreement for tissue specimens* tested with different genotyping methods. (DOC 31 kb) [file 12879_2015_1281_MOESM3_ESM.doc]

**Supplementary table 3. Human papillomavirus (HPV) genotyping agreement for tissue specimens* tested with different genotyping methods**

|  |  |  | SPF-LiPA_25_  vs  Onclarity |  | SPF-LiPA_25_  vs  Inno-LiPA |  | SPF-LiPA_25_  vs  Linear Array |  | Inno-LiPA  vs  Onclarity |  | Inno-LiPA  vs  Linear Array |  | Linear Array  vs  Onclarity |
| --- | --- | --- | --- | --- | --- | --- | --- | --- | --- | --- | --- | --- | --- |
| **Classification of agreement** |  |  | **N (%)** |  | **N (%)** |  | **N (%)** |  | **N (%)** |  | **N (%)** |  | **N (%)** |
| Identical |  |  | 37 (61.7) |  | 44 (73.3) |  | 45 (75.0) |  | 35 (58.3) |  | 45 (75.0) |  | 36 (60.0) |
| Compatible |  |  | 15 (25.0) |  | 8 (13.3) |  | 5 (8.3) |  | 13 (21.7) |  | 12 (20.0) |  | 11 (18.3) |
| Discrepant |  |  | 8 (13.3) |  | 8 (13.3) |  | 10 (16.7) |  | 12 (20.0) |  | 3 (5.0) |  | 13 (21.7) |

* Genotyping agreement for 60 paired formalin-fixed and paraffin embedded (FFPE) specimens in the SUCCEED study; Onclarity, The BD Onclarity™ HPV Assay; Inno-LiPA , The Inno-LiPA system ; Linear Array , The PGMY09/11 Linear Array, and SPF-LiPA_25_ , The SPF_10_-DEIA, LiPA_25_ (version 1); Identical, same number and type identified ; Compatible, at least one type in common identified ; Discrepant, no type in common identified or FFPE sample HPV negative.
